# Evaluation of the Therapeutic Potential of Synthetic Growth Hormone-Releasing Hormone Antagonist MIA-690 as a Cognitive Modulator in a Mouse Model of Gulf War Illness

**DOI:** 10.3390/ijms26178516

**Published:** 2025-09-02

**Authors:** Luis Manuel Salgueiro-Tosta, Arumugam Radhakrishnan Jayakumar, William Kochen, Renzhi Cai, Wei Sha, Erik Johnson, James O’Callaghan, Miklós Jászberényi, Andrew Victor Schally, Nancy Klimas

**Affiliations:** 1 Institute for Neuro Immune Medicine, Nova Southeastern University, Davie, FL 33314, USA; 2 Endocrine, Polypeptide and Cancer Institute, Miami VA Healthcare System, Miami, FL 33125, USA; 3 Department of Obstetrics, Gynecology and Reproductive Sciences, University of Miami Miller School of Medicine, Miami, FL 33136, USA; avrj_2000@yahoo.com; 4 Department of Neuroscience, High Point University, High Point, NC 27262, USA; wkochen@highpoint.edu; 5 U.S. Army Medical Research Institute of Chemical Defense, Aberdeen Proving Ground, MD 21010, USA; 6 Molecular Neurotoxicology Laboratory, Centers for Disease Control, Morgantown, WV 26505, USA; 7 Department of Pathophysiology, University of Szeged, H-6720 Szeged, Hungary

**Keywords:** Gulf War illness, neurodegeneration, neuroinflammation, growth hormone-releasing hormone, hypothalamic–pituitary–adrenal axis, Morris water maze, novel object recognition test, grip strength test, open field test

## Abstract

Gulf War illness (GWI) is a multi-symptom disorder affecting veterans of the Persian Gulf operations. Persistent neuroendocrine dysregulation contributes to impairing cognitive capacity and generates anxiety-like behavior. Effective treatments for this illness are challenging due to compromised metabolism, increased oxidative stress and neuroinflammation, perpetuated by chronic stress and hypothalamic–pituitary–adrenal (HPA) axis dysfunction. This neuroinflammation can be alleviated with synthetic antagonistic analogs of the growth hormone-releasing hormone (GHRH) through modulation of the HPA axis. We evaluated the efficacy of the GHRH antagonist analog, MIA-690, against cognitive impairment and anxiety-like behavior in GWI. Mice exposed to an experimental GWI model involving corticosterone (CORT) and diisopropylfluorophosphate (DFP), followed by CORT and lipopolysaccharide (LPS), received a daily subcutaneous dose of 10 μg of MIA-690 for 10 days. Assessments of spatial memory, recognition capacity, somatic health, anxiety and innate survival were carried out, combining the Morris water maze (MWM), novel object recognition (NORT), grip strength (GST), and open field (OFT) tests. Learning efficiency was selectively enhanced in females using the MWM. There were no significant differences in the recall capacity and performance on the OFT, NOR, and GST tasks. Our findings suggest that the MIA-690 dosage is sufficient to improve learning deficits in experimental GWI exposures.

## 1. Introduction

GWI is defined as an unexplained chronic multi-symptom illness (MUCMI) primarily affecting military personnel deployed to the Persian Gulf theater from 1990 to 1991 [1]. The symptoms in GWI patients are insidious and persistent [2], and the severity of these symptoms correlates with the emergence of neurological signs, which involve asynchronous neural interactions, cognitive impairment, and mental illness [3,4,5]. Unlike other neurological health conditions, GWI patients experience a complex disruption in their normal thinking processes due to dysfunction in the neuronal circuits linked to memory storage and retrieval [6,7]. A high prevalence of post-traumatic stress disorder (PTSD), depression, and anxiety is reported among those deployed during the Persian Gulf War [8]. The levels of anxiety in patients with GWI are higher compared to non-deployed veterans [9]. This burden creates a sustained sense of distress that often leads to various neurological co-morbidities. GWI exemplifies how toxic and environmental exposures can contribute to complex chronic illnesses.

The etiology of the condition is complex, involving neuroinflammatory and neurodegenerative outcomes that simultaneously affect the immune and endocrine systems. Understanding the physiopathology of this association is crucial for developing new treatments to alleviate the mental health challenges faced by veterans of the Persian Gulf War. Due to the chronic presence of high levels of cortisone and Acetylcholine (ACh)—caused by acetylcholinesterase inhibitors (AChEIs) as the aggravating chemical—these patients develop new patterns of regulation that differ from the responses of an intact hormonal and immune signaling network. Both adaptive and innate immune mechanisms are involved in developing and persisting abnormal immune signaling. Despite the aggravating factors, synthetic GHRH analogs (agonistic and antagonistic) exhibit potent immunomodulatory activity on inflammatory messengers, which are causal for diverse pathologies in different organ systems [10,11]. Suppression of growth in various human cancer lines [12,13,14,15,16,17,18] suggests a direct impact on the adaptive immune response [10,19,20]. Additionally, the beneficial effects on cardiovascular remodeling and endothelial support [21,22,23,24], neuronal protection and cognitive function [11,25,26,27,28], chronic kidney disease complications [29,30,31], and lung inflammation and fibrosis [32,33,34] indicate a strong inhibition of innate immunity.

GHRH is a hypothalamic neurohormone that modulates various endocrine and metabolic functions. GHRH exhibits effective trophic activity due to its strong regulation of the components of the GHRH–growth hormone (GH)-insulin-like growth factor (IGF) axis. It stimulates the direct release of GH from the pituitary gland and the subsequent secretion of insulin-like growth factor 1 (IGF-1) in the liver. This hormonal axis facilitates the metabolism of proteins, fats, and carbohydrates and is essential for cell growth, regeneration, and maintaining bodily structure and function [35]. The GHRH-GH-IGF axis plays a direct role in cognitive function [36,37,38,39,40], and abnormal variations in its secretion contribute to the progression of central nervous system (CNS) pathologies [36,37,39,41]. Synthetic analogs of GHRH are bioactive peptides capable of modulating inflammatory pathways, correcting metabolic imbalances, and enhancing endothelial function [42,43,44,45,46]. Therefore, the development of these peptides has attracted interest due to their potential therapeutic applications [35]. Despite advances in studying the modulation of the GHRH axis by synthetic GHRH analogs, there remains a critical gap in our understanding of how the activity of these compounds can help improve cognitive deficiencies in various physiological and pathological conditions. Exogenous agonistic peptides administered directly to the CNS can trigger ultrashort loop feedbacks, explaining their inhibitory effect on memory consolidation [47,48]. Nevertheless, they can be beneficial in subtle age-related cognitive deficits [49,50,51]. Conversely, GHRH antagonists show promise in enhancing learning and memory due to their potent modulatory role in metabolic imbalances mediated by IGF-1 [39,41]. Based on this rationale, Jászberényi et al. (2012) demonstrated that this biological action is also advantageous in delaying the detrimental secretion of IGF in a genomic model of familial Alzheimer’s disease [28].

Existing evidence suggests that the combined effects of exposure to warfare agents and the stressful environment of the Persian Gulf are likely part of the etiologic factors of GWI [52,53,54]. AChEIs are identified as potential etiologic agents in the Persian Gulf exposome [55,56,57]. ACh is a neurotransmitter that plays a multifaceted role in the CNS and is a crucial natural stimulus for cognitive processes. Deficits in the release of ACh, sensory deprivation, or pharmacological blockade of ACh receptors in the basal forebrain led to significant dysregulation of the dynamics of cortical circuits responsible for encoding sensory information and attentional processes (wakefulness, attention, and memory). Conversely, since cholinergic interneurons in the striatum are inhibitory, excessive ACh activity can induce motor impairments related to Parkinsonism. The harmful effects of AChEIs used as warfare agents differ greatly from the drug-induced effects of anticholinergic compounds employed therapeutically for various neurodegenerative and psychiatric disorders. Consilience among silica, preclinical, and clinical studies supports the hypothesis that acute increases in synaptic levels of ACh, combined with stress, play a key role in the initial release of pro-inflammatory mediators. These mediators trigger subsequent oxidative stress and epigenetic changes, desensitizing glucocorticoid receptors (GR) and disrupting IGF-1 levels [58,59]. Studies suggest that this disruption of the GHRH-GH-IGF axis may be linked to a persistent inflammatory cycle, contributing to ongoing cognitive dysfunction in GWI. Therefore, targeting the primary regulator of this axis with a potent GHRH antagonist to modulate IGF levels provides a strong rationale for evaluating this compound as a treatment for GWI. This pre-clinical study aims to evaluate the beneficial effects of the biologically active synthetic GHRH antagonistic analog, MIA-690, on cognitive impairment in an animal model of GWI.

## 2. Results

### 2.1. Morris Water Maze

#### Learning

A total of 51 animals (22 males, 29 females) were used to evaluate the spatial memory using an MWM.

**Figure 1:**
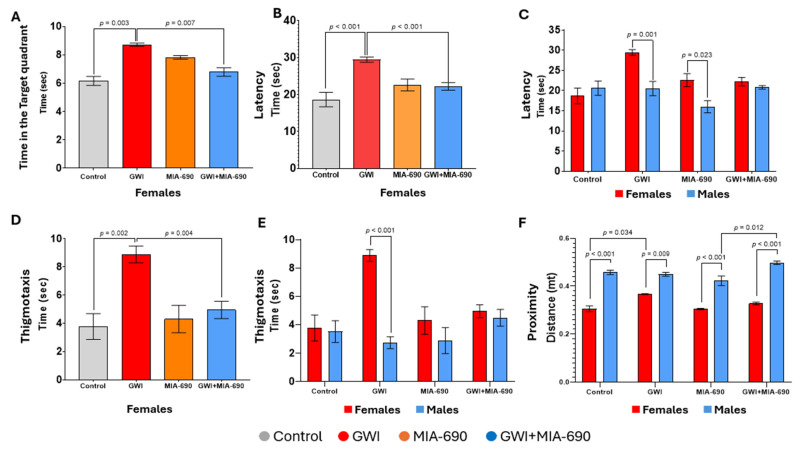
Learning parameters reported for behavioral evaluations using the MWM task following the GWI exposure. Time spent in the target quadrant across the learning days for females (**A**), time (latency) to reach the target for females (**B**), latency for both sexes (**C**), thigmotaxis (distance of the swimming path near the walls while attempting to escape the maze) for females (**D**), thigmotaxis for both sexes (**E**), and proximity or average distance between the animal and the centroid (center of the target) for both sexes (**F**). Values are presented as mean ± SEM. Experimental groups are defined as control (●), GWI-exposed (●), MIA-690 control (●), and GWI + MIA-690 treatment (●). Resultant *p*-values are shown in the graphic. Statistical significance was set at *p* < 0.05. Control, *n* = 10 (5♂ + 5♀); GWI-exposed, *n* = 14 (4♂ + 10♀); MIA-690, *n* = 10 (5♂ + 5♀); GWI + MIA-690, *n* = 17 (8♂ + 9♀).

Time in the target quadrant (Figure 1A): A between-subjects analysis revealed a significant three-way interaction between GWI, MIA-690 treatment, and sex on time spent in the target quadrant for MWM F(1, 43) = 4.841, *p* = 0.033. Pairwise comparisons indicate vehicle females exposed to GWI (GWI-exposed group) spent significantly more time in the target quadrant compared to vehicle control (control group) females (*p* = 0.003) Additionally, females with GWI (GWI-exposed group) spent significantly more time in the target quadrant after vehicle treatment compared to those GWI-exposed females receiving MIA-690 treatment (*p* = 0.007). There were no significant differences between the experimental groups of male mice.

Latency (Figure 1B,C): There was a significant three-way between-subjects interaction effect for GWI, MIA-690 treatment, and sex on the average time spent each day to reach the target F(1, 43) = 9.465, *p* = 0.004. Pairwise comparisons indicate that females exposed to GWI who did not receive treatment (GWI-exposed group) took significantly longer to reach the platform than females from the GWI + MIA-690 group (*p* < 0.001). The females of the GWI-exposed group took significantly longer to find the platform than the control group of females (*p* < 0.001). GWI females took significantly longer to find the platform than GWI males (*p* = 0.001). Finally, MIA-690 control females took significantly longer to find the platform than MIA-690 control males (*p* = 0.023) Male mice did not show any significant interaction between groups.

Thigmotaxis behavior (Figure 1D,E): A between-subjects analysis revealed a significant three-way interaction between sex, GWI exposure, and MIA-690 treatment on thigmotaxis behavior (i.e., the tendency to swim close to the walls of the pool), F(1, 43) = 4.242, *p* = 0.043. Pairwise comparisons indicate that the GWI-exposed females had significantly increased thigmotaxis over control females (*p* = 0.002). Females exposed to GWI and treated with vehicle (GWI-exposed group) spent significantly more time (hugging) swimming near the pool walls compared to those treated with MIA-690 (*p* = 0.004), indicating increased thigmotaxis behavior. Untreated GWI-exposed females spent significantly more time along the walls than untreated males with GWI (*p* < 0.001).

Average proximity (Figure 1F): There was a trending three-way between-subjects interaction for sex, GWI, and MIA-690 for proximity F(1, 43) = 4.069, *p* = 0.050. Pairwise comparisons indicate a significant effect of females who were exposed to GWI (GWI-exposed) with significantly higher proximity than control females (*p* = 0.034). This indicates that exposure to GWI increases the time that the animal spends farther away from the target. Males exposed to GWI and treated with MIA-690 (GWI + MIA-690 male group) had significantly higher proximity than MIA-690 control males (*p* = 0.012), suggesting that MIA-690 treatment did not fully mitigate spatial memory deficits in GWI-exposed males. Across all treatment and conditions, male mice (both control and MIA-690 groups, as well as GWI-exposed and GWI + MIA-690-treated mice) exhibited significantly higher proximity scores compared to their female counterparts (*p* < 0.01), indicating a sex-dependent difference in spatial memory performance.

Random swimming behavior was assessed by measuring the time spent in the opposite quadrant. Although GWI-exposed females spent more time in the target quadrant, which might suggest improved performance (Figure 1A), this group also exhibited increased latency (Figure 1B,C) and thigmotaxis (Figure 1D,E), indicating lack of engagement with memory-directed search and difficulty in the efficient location of the platform. In contrast, GWI + MIA-690 females, despite spending less time in the target quadrant, reached the platform faster (Figure 1B–D), suggesting that familiarity with this task enabled more effective use of spatial memory and a goal-directed search strategy.

### 2.2. Novel Object Recognition

**Figure 2:**
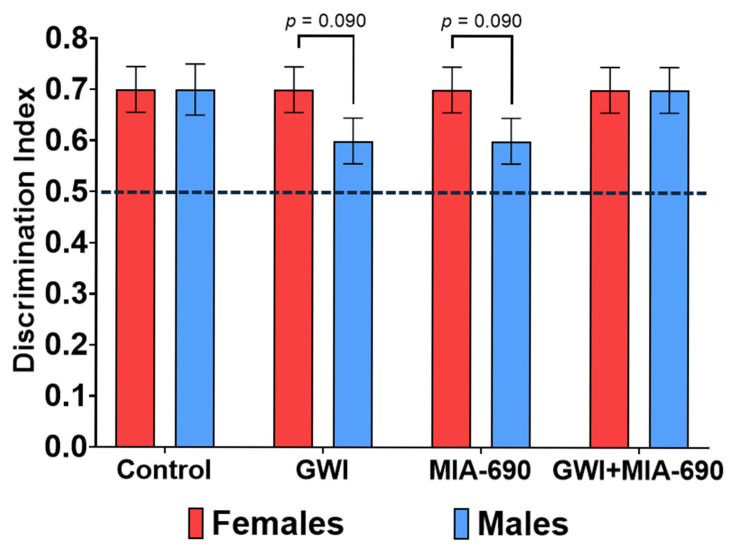
Evaluation of the pro-cognitive effect of MIA-690 on animals exposed to the experimental CORT/DFP-CORT/LPS model of GWI. Recognition memory performance in male and female C57BL/6J mice was assessed with the discrimination index (time spent exploring the novel object/total time spent exploring both objects) across experimental groups. All groups showed indices above 0.5, indicating recognition of the novel object. A trending effect of sex was observed, with females showing higher discrimination indices than males (*p* = 0.090). No significant differences were found between treatment groups. Values are presented as mean ± SEM. Resultant *p*-values are shown in the graphic. Control, *n* = 9 (4♂ + 5♀); GWI-exposed, *n* = 10 (5♂ + 5♀); MIA-690, *n* = 10 (5♂ + 5♀); GWI + MIA-690, *n* = 10 (5♂ + 5♀).

A total of 39 C57BL/6J mice (19 males and 20 females) were evaluated using the NORT. All experimental groups demonstrated a positive discrimination index (>0.5) 24 h post familiarization, indicating intact recognition memory across conditions. Statistical analysis revealed a trending effect of sex on the discrimination index (F(1, 31) = 3.054, *p* = 0.090), with females exhibiting a higher index than males (*p* = 0.09), suggesting a potential sex-related influence on recognition memory performance. However, no statistically significant differences were observed between the experimental groups. GWI + MIA-690 individuals did not show significant improvement in recognition memory compared to GWI-exposed animals (Figure 2). These findings indicate that, under the conditions tested, MIA-690 did not exert a measurable pro-cognitive effect in the NORT paradigm.

### 2.3. Open Field Test

**Figure 3:**
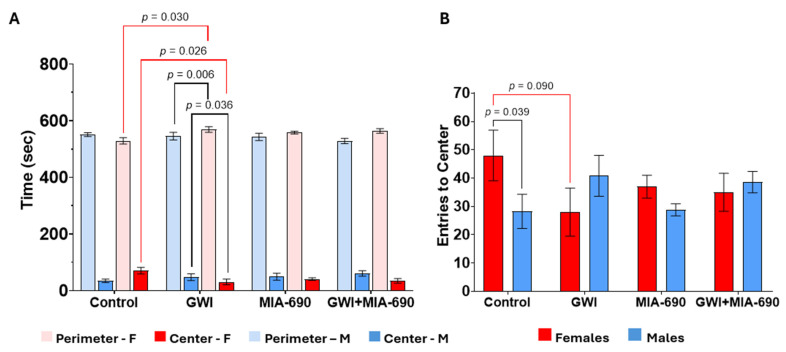
Exploratory behavior in GWI-exposed animals. The OFT measured as time spent in the perimeter and center zones (**A**), and number of entries into the center zone (**B**). MIA-690 treatment did not significantly affect exploratory behavior. Significant differences were observed in females (red significance bars) and in sex comparisons (black significance bars) as a consequence of the GWI exposure. Values are presented as mean ± SEM. Resultant *p*-values are shown in the graphic. Control, *n* = 9 (4♂ + 5♀); GWI-exposed, *n* = 10 (5♂ + 5♀); MIA-690, *n* = 10 (5♂ + 5♀); GWI + MIA-690, *n* = 10 (5♂ + 5♀).

A total of 19 male and 20 female C57BL/6J mice were used for the OFT. The exploratory behavior of the animals was significantly diminished in those exposed to the experimental CORT/DFP-CORT/LPS GWI model (Figure 3). There was a significant interaction effect of sex and GWI for time spent at the perimeter (F(1, 31) = 5.137, *p* = 0.031), number of entries to the center box (F(1, 31) = 6.243, *p* = 0.018), and total amount of time spent in the center (F(1, 31) = 6.054, *p* = 0.020). Pairwise comparisons showed that control females spent significantly more time in the center (*p* = 0.026) and spent less time at the perimeter (*p* = 0.030), and there was a trending effect of more center entries (*p* = 0.090) than GWI females. Additionally, GWI males spent significantly more time in the center of the box than GWI females (*p* = 0.036) and less time in the perimeter of the box (*p* = 0.006). Control males made significantly less entries to the center of the box than control females *p* = 0.039.

### 2.4. Grip Strength Test Results

**Figure 4:**
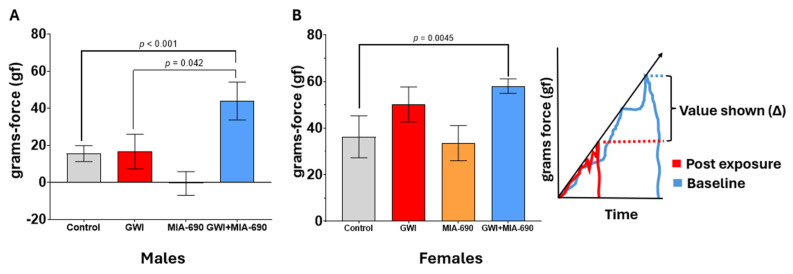
Evaluation of GST. Animals were assessed using a grip strength meter. Average peak force variation (grip strength at baseline—grip strength after exposure) from three trials per day for males (**A**), and females (**B**). Values are presented as mean ± SEM. Experimental groups are defined as control (●), GWI-exposed (●), MIA-690 control (●), and GWI + MIA-690 treatment (●). Resultant *p*-values are shown in the graphic. Statistical significance was set at *p* < 0.05. Control, *n* = 10 (5♂ + 5♀); GWI-exposed, *n* = 14 (4♂ + 10♀); MIA-690, *n* = 10 (5♂ + 5♀); GWI + MIA-690, *n* = 17 (8♂ + 9♀).

Strength capacity and cognitive function are related, with diminished strength indicating subtle, progressive cognitive decline (Figure 4). Handgrip strength can be used to assess cognitive status in aging populations, and some authors suggest monitoring strength capacity in patients with cognitive impairment [60]. Clinical evidence supports the use of the GST for evaluating cognitive problems in the elderly due to its predictive value [60,61,62,63,64,65]. We used the outcomes of this task as an indirect evaluation of CNS health. A total of 51 animals (22 males, 29 females) were used. There was a significant interaction effect of GWI and MIA-690 on change in grip strength before and after the CORT/DFP-CORT/LPS exposure (F(1, 43) = 4.657, *p* = 0.037).

GWI exposure affected the mice, causing a significative increase in the variation of the grip strength (F(1, 43) = 11.230, *p* = 0.002). There was also a significant main effect of sex on grip strength (F(1, 43) = 16.780, *p* < 0.001) without any significant effect of the MIA-690 treatment (F(1, 43) = 0.425, *p* = 0.518).

Pairwise comparison show significant sex-based differences within controls (*p* = 0.006), GWI-exposed groups (*p* = 0.005), MIA-690 controls (*p* = 0.005), and GWI + MIA-690 (*p* = 0.007). A significant compounding effect was observed due to the interaction between treatment × exposure × sex, where GWI + MIA-690 males (*p* < 0.001) and GWI + MIA-690 females (*p* = 0.045) differed from the corresponding controls (Figure 4).

GWI-exposed male mice had significantly higher grip strength after MIA-690 treatment (GWI + MIA-690 group) compared to the untreated (GWI-exposed) animals (*p* = 0.042). This effect was not observed for females.

## 3. Discussion

Previous studies have shown that the synthetic GHRH antagonistic analog, MIA-690, potentiates the GHRH-GH-IGF axis. The complex nature of this neuroendocrine pathway allows the endocrine intervention of numerous physiologic and pathologic processes, including aging-related learning impairments and the progress of Alzheimer’s disease, a chronic neurological disorder with severe cognitive deficits [28,66,67]. A protective anti-inflammatory effect of synthetic GHRH antagonistic analogs has been reported in several models of cancerous and non-cancerous tissues [10,12,14,35]. Recruitment of p53 is an important suppressor of iNOS, COX-2, and active NF-κB [35,66,68,69,70,71]. Concurrent modulation of these inflammatory messengers and neuropeptide signaling justify the evaluation of the beneficial effect of the MIA-690 in the CNS.

Our present evaluation of the compound MIA-690 revealed no significant impact on the adverse changes in exploratory behavior, recognition memory, behavioral despair, or strength capacity—key domains adversely affected in the experimental CORT/DFP-CORT/LPS model of GWI. Despite this limitation, the compound demonstrated a selective and moderate efficacy in enhancing learning parameters in female subjects.

MIA-690 does not antagonize the lethality of organophosphate-induced cholinergic crisis, suggesting that its cognitive benefits may depend on different molecular mechanisms. Previous studies using sequential exposure to CORT-DFP in mice demonstrated that CORT primes the CNS to produce heightened neuroinflammatory responses to the neurotoxicant, DFP, favoring persistent allostasis with minimal systemic inflammation contribution [72,73,74,75,76,77]. Corresponding studies and clinical reports show that increased inflammatory responses and epigenetic programming are linked to cognitive impairment [6,78,79,80,81,82,83,84,85,86]. Acute organophosphate exposures can induce epigenetic alterations, impede neuronal resilience, and promote allostasis, cellular senescence, and cognitive dysfunction [6,87,88]. This study minimized the risks of cholinergic toxicity while maintaining the minimal DFP dosage to ensure ample circulating toxicant to interact with messenger and regulatory proteins. This allowed the evaluation of the cognitive capacity using several behavioral tasks.

We investigated the effects of MIA-690 treatment on spatial learning and memory in male and female mice using the MWM task. Female mice demonstrated significant improvements in learning parameters following treatment, whereas male mice showed no such responsiveness. We used the time spent in the opposite quadrant as a control measure to assess random swimming behavior and memory deficiency. This parameter was interpreted alongside complementary parameters. When interpreted independently, reduced time in the target quadrant reflects impaired spatial memory. However, although GWI-exposed females spent more time in the target quadrant—suggesting better performance—this group also exhibited increased latency, thigmotaxis, and proximity, indicating difficulty in locating the platform. In contrast, GWI + MIA-690 animals that spent less time in the quadrant reached the target more efficiently, suggesting active use of spatial memory and a more effective search strategy.

Interestingly, males across all experimental groups consistently exhibited higher proximity values compared to females and did not show significant differences in latency, time spent in the target quadrant, or thigmotaxis. This behavior suggests a potentially impaired or limited spatial encoding, which may partly explain their lack of responsiveness to MIA-690, as the treatment may require a baseline level of cognitive function or hippocampal integrity to exert its effects. Additionally, the sex-dependent pattern may reflect intrinsic behavioral or neurobiological differences in spatial search strategies, which become apparent under the stress induced by GWI exposure. Another consideration is the dosage of MIA-690, which proved counterproductive for cognitive performance due to a threshold-dependent antagonistic effect. Excessive inhibition of the GHRH-GH-IGF axis, particularly under conditions of GWI-induced hormonal dysregulation, may increase the susceptibility of male mice to altered neuroendocrine signaling, thereby influencing spatial memory. These paradoxical findings underscore the importance of considering both sex and dosage as critical variables to avoid counterproductive outcomes in hormonal approaches to neuroinflammation. Supporting this interpretation, thigmotaxis behavior revealed a significant interaction between sex, GWI exposure, and treatment. While untreated GWI-exposed females showed elevated wall-following behavior, MIA-690 significantly reduced this anxiety-like response. In contrast, the effect of MIA-690 on thigmotaxis in males appeared masked, suggesting that although the treatment may exert anxiolytic-like effects, these are less detectable due to baseline behavioral variability or stress-induced interference. Additional factors may contribute to the observed sex-specific differences, including differential bioavailability, sex-dependent pharmacokinetics and pharmacodynamics, the neuroprotective effects of hormonal regulation in females, or sex-driven responses to stress and anxiety. Male mice may have experienced higher stress levels during the MWM task, leading to impaired learning and elevated proximity values. In contrast, the greater adaptability of females to the testing environment may have allowed for more effective engagement with the task and a greater benefit from the treatment.

Somatic health at the CNS was evaluated indirectly using parameters measured with the GST [65,89,90]. The group of animals treated with MIA-690 did not maintain their grip strength. They exhibited higher peak forces than the exposed “untreated” group, indicating a decline in strength and suggesting a lack of support of the somatotropic axis. This suggests that MIA-690 did not aid cellular health beyond its antagonistic action on the GHRH-GH-IGF axis.

Additional testing of adaptive stress responses was conducted using OFT (anxiety). Results observed in the OFT demonstrated several patterns. Control females exhibited greater exploratory behavior, as indicated by more time spent in the center and less time at the perimeter, with a trend toward more center entries compared to the group of GWI-exposed females. In contrast, GWI females showed more anxiety-like behavior. Among GWI-exposed animals, males were less anxious than females, spending more time in the center and less at the perimeter. Interestingly, control males made fewer center entries than control females, which may reflect sex differences in exploratory drive or anxiety levels within the control group. Overall, these findings on the OFT suggest that both GWI and sex influence anxiety-like behavior and exploratory patterns. OFT did not show any advantage in detecting the benefits of MIA-690 over the parameters of despair or anxiety.

These results are not consistent with previous reports by Recinella et al. (2020, 2021), which demonstrated that a group of potent analogs of the MIA-series of GHRH antagonists, MIA-602 and MIA-690, exert anxiolytic and antidepressant-like effects in mice [20,91]. Their study employed a combination of behavioral tasks resembling PTSD (the home cage locomotor activity test, OFT, light–dark box test, and tail suspension test), alongside ex vivo exposure of tissue specimens from the prefrontal cortex and hippocampus. They reported activation of Nrf2 and BDNF/TrkB pathways as the main mechanisms of action, along with reductions in oxidative stress and pro-inflammatory cytokines—mechanisms implicated in GWI-related cognitive and emotional impairments [20,91]. However, this discrepancy may be attributed to differences in the experimental design. Notably, Recinella et al. [20,91] did not incorporate any toxicant exposure such as CNS priming with CORT/DFP in their behavioral experiments. Their mechanistic analysis was conducted entirely ex vivo, using tissue specimens treated with LPS as the primary toxicant. This approach does not replicate the complex neuroimmune environment present in vivo following GWI-relevant exposures, which may explain the divergent outcomes observed with these GHRH analogs.

GHRH generates critical biological responses at diverse autocrine, paracrine, and endocrine levels [35,71,92,93,94]. The functional activity of the hormone released by the hypothalamus occurs through its direct interaction with extra- pituitary receptors in target tissues or by promoting an indirect trophic effect through the release of GH from the anterior pituitary and IGF-1 from secretory cells. Thus, the modulation of cognitive processes using GHRH or GHRH analogs can occur by intervening in the GHRH-GH-IGF axis. Discrepancies in the results obtained from evaluating GHRH and its synthetic analogs can be understood by considering each study’ s respective physiological and pathophysiological context. In healthy young men, the intranasal administration of exogenous GHRH_1–44_ resulted in abnormal hippocampal memory consolidation [48]. Similarly, the cerebroventricular injection of the same agonist triggered a negative ultrashort loop feedback signal, decreasing the neurosecretion and release of GH in male rats [47]. The normal aging process is associated with subtle cognitive declines due to deregulated nutrient sensing, deficient intercellular communications, stem cell exhaustion, and functional brain alterations [95]. These deficits were resolved using recombinant GHRH to stimulate the frail responses of the GHRH-GH-IGF axis [49,50,51,96]. Failures of the hypothalamic–pituitary–target axis can be attributed to epigenetic modifications and reduced proteostasis, leading to the shrinkage and atrophy of the target organs [97,98]. A consequent hyperactivity of the hypothalamus occurs due to faint or missing negative feedback. Consistent reports indicate that GHRH antagonists have a positive impact on the learning and memory responses of these elderly cohorts [38,39,40,99]. This effect can be explained by an inhibition of metabolism, comparable to long-term energy intake restrictions. A moderate reduction in GH and IGF-1 influences metabolic processes and helps to delay age-related morbid events [99,100,101,102,103].

Individuals with acquired neurological conditions develop inefficient, persistent, and harmful allostatic responses that exceed mild cognitive declines [4,104,105,106,107]. Synthetic GHRH antagonistic analogs can alleviate cognitive declines caused by acute exposure to toxicants or degenerative models of Alzheimer’s disease (AD). Jászberényi et al. (2012) demonstrated the therapeutic potential of MIA-690 in mice of the 5xFAD strain, a degenerative model showing cognitive and somatic deficits due to multiple mutations of incomplete penetrance [28].

Our study showed a limited and selective beneficial effect on the learning patterns of animals exposed to the acute effects of GWI toxicants, where cognitive impairment is perpetuated by identifiable epigenetic modifications [57,80,108,109,110]. Although GWI shares key pathological features with AD—including chronic neuroinflammation, metabolic dysfunction, and dysregulation of the GHRH-GH-IGF axis—our findings suggest that the mechanisms underlying the neuroprotective effects differ. Rather than supporting the idea that GHRH antagonists offer broad neuroprotective benefits across mechanistically related conditions, our results point to a more condition-specific mode of action [66,67].

This initial study was limited to the behavioral responses of the affected and treated animals. We demonstrated a beneficial modulatory effect of MIA-690 on the learning capacity of the animals. Nevertheless, the compound was not effective in restoring normal exploratory patterns, maintaining an active recognition capacity, or reducing the exhaustion of survival instincts resulting from exposure to GWI toxicants. An adverse effect was even observed in the ability of the animals to maintain their grip strength. To elucidate the mechanisms underlying both the beneficial and potentially adverse effects of the analog as an alternative treatment for GWI, it is imperative to conduct complementary experiments involving biomarkers. One challenge encountered was the inconsistency in molecular (non-genomic) biomarker data, possibly due to the rapid inflammatory events triggered by the acute exposure or the instability of the molecular messengers involved. In this context, genomic and silica approaches become invaluable tools for future studies, guiding the design of new experiments and models to address specific molecular and mechanistic questions.

Alternative models that can demonstrate associations between behavioral and biochemical data related to potential molecular targets should also be considered. The various pharmacological targets linked to the direct and trophic actions of GHRH and the GHRH-GH-IGF axis enable the exploration of a wide range of mechanistic alternatives involved in the beneficial effects of the antagonistic peptides. A notable example is the direct impact on the liver and the associated metabolic pathways that, when modulated, can influence neuronal activity and improve cognitive pathology [111,112].

Future research should refine and complement this task to establish a more robust association with cognitive function or appropriate CNS health biomarkers. Significant effort was dedicated to determining the proper dosages of DFP and LPS, which helped to avoid the lethal and cardiovascular effects of DFP as well as signs of hypoperfusion induced by LPS due to septic shock in the CNS or other tissues. Future behavioral or longitudinal studies must pay close attention to these parameters and variables. Studies differentiating the effects of anxiety from direct cognitive mechanisms and their relevance for treating GWI patients are imperative. Further preclinical studies are needed to discern the beneficial effect of GHRH-GH-IGF axis modulation on cognitive deficits and anxiolytic activity in GWI [101,113]. Specific mechanisms of action for the MIA-690 must be elucidated, focusing on the coordinated epigenetic and molecular patterns influencing cognition and the protective effect on long-term memory and memory consolidation [114].

We demonstrated that MIA-690 treatment selectively enhanced learning patterns, with a preferential effect observed in female subjects using the MWM task. However, no significant improvements were detected in the treated groups (GWI + MIA-690) across other behavioral tasks assessing exploratory behavior, recognition memory, or behavioral despair. Notably, the GST revealed no beneficial effect of the treatment. Instead, a reduction in strength was observed when the MIA-690 treatment followed GWI exposure. This effect was sex-dependent, showing a weak trend in females and reaching statistical significance in males. This counterproductive outcome suggests predominance of the inhibitory effect of MIA-690 on the GHRH-GH-IGF axis. Deficiencies in this axis can lead to reduced protein synthesis, which is essential for muscle maintenance and the integrity and excitability of the neuromuscular junction. A consequent deficiency in the recruitment of motor units may explain the reduction in the ability to generate force during the grip strength task. Further studies employing different behavioral parameters and robust cognitive assessments are required to evaluate the therapeutic potential of synthetic GHRH antagonistic analogs to improve behavioral deficits in patients afflicted by GWI.

## 4. Materials and Methods

### 4.1. Peptide

The synthetic GHRH antagonist analog, MIA-690 (Table 1), was provided by the Endocrine, Polypeptide, and Cancer Institute laboratory at the Miami VA Healthcare System in Miami, FL. The peptide was synthesized using standard solid-phase methods and purified through reverse-phase high-pressure liquid chromatography (Mettler-Toledo Rainin, LLC, Oakland, CA, USA) [43]. After purification, the purity of the peptides was examined using analytical HPLC (Hewlett-Packard, Spring, TX, USA). Final fractions of the peptide with at least 95% purity were lyophilized, and their molecular masses were confirmed by matrix-assisted laser desorption/ionization time-of-flight mass spectrometry (autoflex^®^ maX, Bruker Corporation, Fitchburg, WI, USA) before dilution for dosing. The lyophilized peptide was dissolved in 0.1% DMSO (Sigma) and 10% propylene glycol (Sigma-Aldrich, St. Louis, MO, USA). The synthesis process ensured safety and pharmaceutical grade standards. Additionally, pooled samples of the purified peptide were verified to be free of biological contaminants using polymerase chain reaction and culture methods before administration to the experimental animals.

### 4.2. Chemicals

Analytical-grade reagents were sourced from various commercial suppliers: DFP and LPS (Sigma-Aldrich, St. Louis, MO, USA), CORT or 11β,21-Dihydroxy-4-pregnene-3,20-dione (Steraloids, Inc., Newport, RI, USA), and Electrogel, a support gel containing electrolytes (Bio-Serv^®^, Prospect, CT, USA).

### 4.3. Animals

Female and male mice of the C57BL/6J strain, aged 7 to 8 weeks, were purchased from Jackson Labs (Bar Harbor, ME, USA). Upon arrival, the animals were housed in a temperature-controlled room (21 ± 1 °C) in sterile cages with filtered positive-pressure ventilation in a 12 h light/12 h dark cycle. They were fed autoclaved chow and water ad libitum. All procedures were conducted under the guidelines of the Declaration of Helsinki and the Miami VAHCS Institutional Animal Care and Use Committee (protocol number 0137.02, approved on 13 June 2017). Compliance with the ARRIVE guidelines on animal research ensured rigorous and transparent reporting of the experimental outcomes.

The animals were randomly divided into four experimental groups for each test: Group 1 (Control) was a vehicle solution, with no exposure; Group 2 (MIA-690 control) was treated only with MIA-690 (10 µg per animal for ten days), with no exposure; Group 3 (GWI) was not treated, with GWI exposure; and Group 4 (GWI + MIA-690) had GWI exposure with concurrent treatment with MIA-690 (10 µg for ten days, starting four days before the LPS exposure). After attrition, 51 adult mice (22 males, 29 females) were used for spatial memory experiments employing the MWM and GST. A total of 39 mice (19 males, 20 females) were used for OFT and NOR (Table 2).

### 4.4. Experimental GWI Exposure (CORT/DFP-CORT/LPS)

**Figure 5:**
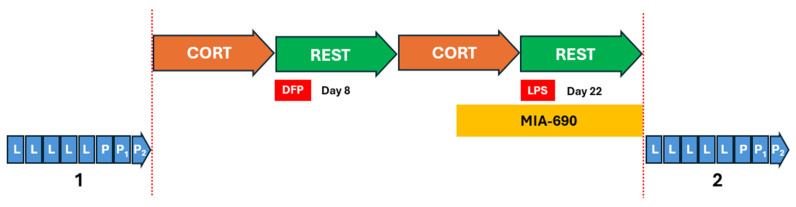
Timeline of the study and sequence of the experimental CORT/DFP-CORT/LPS exposure protocol. The figure details the combined use of CORT, DFP, and LPS. Timing of GWI exposure: CORT (200 mg/Lt, ad libitum, in the drinking water, every other week) (orange arrows); DFP on day 8 (escalated administration starting at 2 mg/kg i.p.), and LPS on day 22 (0.05–0.1 mg/kg s.c.). The yellow strip indicates the treatment period with the GHRH analog, MIA-690 (10 μg/day for 10 days). Resting periods between CORT administration (green arrows). The two time points for behavioral experiments, baseline (1) and after experimental GWI exposure (2), are shown (blue segmented arrows). L: Learning day; P: Probe; P_1_ and P_2_: Extended probe days assessment (48 and 72 h after LPS administration).

The combination of CORT, DFP, and LPS was used to recapitulate the recurring chronic symptoms associated with GWI. After the initial allocation and baseline tests, the following systematic sequence was completed to obtain a persistent heightened neuroinflammatory response comparable to sickness behavior (Figure 5): Mice were exposed to CORT (200 mg/L in 1.2% EtOH, ad libitum in the drinking water) for 7 days. This aims to achieve physiological stress levels simulating the stress of combat [73,115].

Following the CORT administration, a sub-lethal DFP dose was given to induce neuroinflammation [73,74,116]. The DFP dosage was escalated (starting dose, 2 mg/kg, i.p.) until the animal showed non-lethal signs of cholinergic toxicity or the dose of 4 mg/kg was achieved (Table 3). This minimized DFP lethality without limiting the physical capacity of the animals for the behavioral evaluation. Combining CORT and DFP induced a hyper-reactive neuroinflammatory state in the CNS.

After seven days of rest, the administration of CORT (using the same dosage) was repeated. Maintained CORT levels exacerbate the effects of any potential pro-inflammatory stimuli [57,117,118,119].

A single non-septic dose of LPS (0.05–0.1 mg/kg s.c.) was used to trigger a robust neuroinflammatory response to achieve persistence of signs of cognitive impairment.

### 4.5. Titration of Lethal Doses 50 (LD_50_)

**Figure 6:**
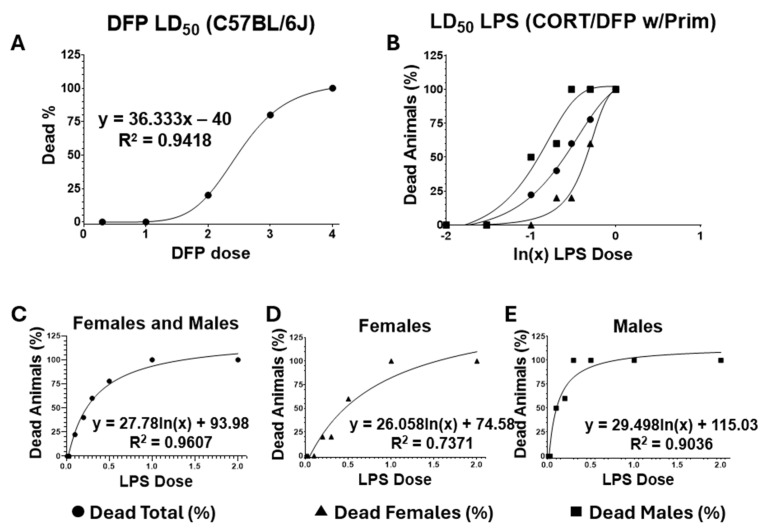
Working doses of toxicants used in the CORT/DFP-CORT/LPS experimental model of GWI. This figure summarizes the dose–response studies conducted to determine the LD_50_ of DFP and LPS. The curves were utilized to interpolate the estimated reference concentrations for administering the compounds. (**A**) The dose–response curve for DFP was established by administering five intraperitoneal (i.p.) doses of (0.3, 1.0, 2.0, 3.0, and 4.0 mg/kg). The determined LD_50_ for i.p. administration of DFP was 2.5 mg/kg. (**B**–**E**) The dose–response curve for LPS in animals previously primed with CORT/DFP was developed by administering six subcutaneous (s.c.) doses of LPS (0, 50, 100, 300, 500, and 1000 μg/kg). The established s.c. LD_50_ for LPS was 0.389 μg/kg (for females), 0.110 μg/kg (for males), and 0.205 μg/kg (combined). These reference values effectively ensured the survival of the mice and the persistence of circulating DFP to produce non-lethal AChE inhibition (cholinergic toxicity)-dependent effects on the exposed animals.

The administration of both DFP and LPS is a crucial step that impacts the overall health of the mice, potentially affecting the evaluation of any behavioral tasks. Therefore, we first calculated the LD_50_ for both DFP and LPS (Figure 6). This allowed for the guided administration of these compounds without compromising the survival and the ability of the animals to complete the designated behavioral tasks.

### 4.6. Exposure to the Toxicants

The mice were weighed before exposure to toxicants. For DFP exposure, we determined the LD_50_ for DFP (2.5 mg/kg) and established a safe dosage for this agent. After the initial CORT administration, the DFP exposure began under the estimated LD_50_, with the intraperitoneal administration of 2 mg/kg of DFP. The dose was gradually increased by 10% of the initial amount every ten minutes until non-lethal signs of AChE inhibition were observed or the dose of 4 mg/kg was reached.

After the resting period, the animals were re-exposed to the ad libitum dose of CORT. This chronic exposure to CORT enhances the neuroinflammatory and neurotoxic responses to non-septic doses of LPS. For LPS exposure, the single subcutaneous administration of LPS (0.05–0.1 μg/kg) constituted half of the calculated LD_50_ (Figure 6, Table 3).

### 4.7. Supportive Care

All mice received subcutaneous fluids (Ringer lactate), warming (37 °C for 1–2 h), and a gel diet for 24–48 h post GWI exposure. The combination of a defined toxicity profile, escalate dosage regimen, and supportive care improved survival and enabled reliable behavioral testing.

### 4.8. Behavioral Tasks

#### 4.8.1. Morris Water Maze (Spatial Memory Evaluation)

Cognitive functioning was evaluated using a comprehensive neuropsychological assessment before and after the CORT/DFP-CORT/LPS GWI exposure model. The MWM consisted of a pool 160 cm diameter and 60 cm in height (Maze Engineers, Skokie, IL, USA) filled with water opaqued with non-toxic white tempera (Blick Art Materials, Galesburg, IL, USA) at 22 ± 2 °C. The maze was divided into four quadrants, with a submerged goal platform in one quadrant. Additional concentric circles aided in analyzing swimming paths and memory usage [120,121]. ANY-maze video tracking system software, version 7.10 (Stoelting Co., Wood Dale, IL, USA), coupled with a digital USB camera (The Imaging Source, Charlotte, NC, USA) and varifocal lens (Computar, Commack, NY, USA), was used to acquire and quantify the mice’s performance. A preliminary cued test assessed swimming speed, motivation, and tendency to float, excluding floaters from the study.

The task consisted of a learning session over 5 consecutive training days, each with 4 trials in which mice searched for a hidden platform. Spatial memory was assessed during a probe session conducted on days 6, 7, and 8, during which the platform was removed, and the mice were allowed to search for 60 s. The parameters recorded during training included escape latency, path length, cumulative distance (CD), and average proximity (PA). For probes, the parameters recorded were CD, PA, platform crossings (PCs), entries to the platform quadrant (EPQs), path length in the platform quadrant (PPQ), and time spent in the platform quadrant (TPQ) [120,121,122]. The platform was relocated for the second learning cycle after LPS administration [120,121,122].

#### 4.8.2. Novel Object Recognition Test (Recognition Memory Task)

We assessed the recognition memory of experimental animals using the NORT, which involves exposing rodents to novel objects or environments to elicit exploratory behaviors [123,124]. The NORT apparatus was a 42 × 42 × 42 cm PVC open-field arena (Maze Engineers, Skokie, IL, USA) with a luminosity of 15 lux to minimize stress and ensure proper video tracking of movements. ANY-maze software and a USB video camera were used to monitor exploration patterns. The NORT task included three sessions: a habituation session without objects (10 min), a familiarization session 20 h later where mice explored two identical objects (10 min), and a test session 6 h after familiarization in which one familiar object was replaced with a novel one (10 min). The parameters analyzed were the difference score (time spent on novel vs. familiar objects) and the discrimination ratio (time on novel object/total exploration time), both indicating recognition memory [123,124].

#### 4.8.3. Grip Strength Test

Forelimb grip strength was measured using a modified version of the conventional GST [125] with a digital grip strength meter (Ugo Basile SRL, Gemonio, Italy). The peak pull force in grams was recorded on a digital force transducer. Each mouse was allowed to grasp a tension bar attached to the transducer, which was mounted in a metal block fixed to a base plate. The base was rotated vertically and kept immobilized. Once the mice grasped the bar, their tails were pulled down until the pulling force exceeded their grip strength. Three trials were conducted per test for each animal, and the mean grip strength (measured in peak force grams of resistance ± SEM) was calculated. Trials were excluded if only one forepaw or the hind limbs were used, if the mouse turned during the pull, or if the mouse left the bar without resistance.

#### 4.8.4. Adaptive Stress Responses

##### Open Field Test (Exploratory Behavior)

The test assessed the anxiety, exploratory behavior, and general activity of mice in a 42 × 42 × 42 cm PVC arena (Maze Engineers, Skokie, IL, USA) [126]. Monitoring and recording of behaviors were conducted using a digital grid set up with the ANY-maze video tracking system. Mice were acclimated for 30 min under consistent lighting (150–200 lux). Each mouse was tested individually in the arena for 10 min. Parameters analyzed included number of entrances, distance traveled, and time spent in different zones (center or periphery) of the arena.

### 4.9. Statistics

A three-way factorial ANOVA (2 × 2 × 2) was conducted on the full dataset to examine the effects of the independent variables on a single dependent variable. For the learning data, a four-way mixed-effects ANOVA (2 × 2 × 2 × 5) was performed to assess both within- and between-subject effects over time. When significant interaction effects were observed, post hoc pairwise comparisons using estimated marginal means testing with least significant difference adjustments were conducted to explore specific group differences. Results are reported as mean ± standard error of the mean (SEM), and statistical significance was set at *p* < 0.05. All analyses were conducted using IBM SPSS Statistics, Version 30.

## Figures and Tables

**Table 1:** Peptide structure. Comparison of the amino acid sequence of the growth hormone-releasing hormone (1−29)-NH2 and the synthetic GHRH antagonist MIA-690.

	Peptides															
	Position of Amino Acid Residues															
	0	1	2	6	8	9	10	11	12	15	20	21	27	28	29	30
hGHGH *	H	Tyr	Ala	Phe	Asn	Ser	Tyr	Arg	Lys	Gly	Arg	Lys	Met	Ser	Arg	NH_2_
MIA-690 **	PhAc-Ada	Tyr	D-Arg	Cpa	Ala	Har	Fpa_5_	His	Orn	Abu	His	Orn	Nle	D-Arg	Har	NH_2_

* hGHRH: human growth hormone-releasing hormone. ** Noncoded amino acid residues and acyl groups used in the synthesis of the GHRH antagonistic analog, MIA-690, are abbreviated as follows. Abu: α-aminobutyric acid; Ada: 12-aminododecanoic acid; Cpa: parachloro-phenylalanine; Fpa_5_: pentafluoro-phenylalanine; Har: homoarginine; Nle: norleucine; Orn: ornithine; PhAc: phenylacetyl.

**Table 2:** Study cohorts, the number of animals allocated per group, and the total numbers in each experiment.

	MWM and GST	NORT and OFT
Vehicle control	10 (5♂ + 5♀)	9 (4♂ + 5♀)
MIA-690 control	14 (10♂ + 4♀)	10 (5♂ + 5♀)
GWI	10 (5♂ + 5♀)	10 (5♂ + 5♀)
GWI + MIA-690	17 (8♂ + 9♀)	10 (5♂ + 5♀)
Total number	51 (22♂ + 29♀)	39 (19♂ + 20♀)

♀: Females; ♂: Males; MWM: Morris water maze; NORT: Novel object recognition test; GST: Grip strength test; OFT: Open field test.

**Table 3:** Toxicological profile and dosage regimen of DFP and LPS.

Toxicant	DFP	LPS
Administration route	i.p.	s.c.
LD_50_	2.5 mg/kg (Males and Females)	0.389 μg/kg (Females), 0.110 μg/kg (Males) 0.205 μg/kg (combined).
* Dose used	Starting: 2.0 mg/kg Maximal: 4.0 mg/kg	0.05 (Males)–0.1 μg/kg (Females)
Dosage regime	Escalated injections 10% increments every 10 min	Single injection
Monitored signs of toxicity	Non-lethal signs: Mild dyspnea, general distress, mild lethargy (hypoactivity, hunched posture), moderate trembling, loss of hiding behavior, moderate colic, and gastrointestinal upset (arched tail, tenesmus, hyper-defecation). Severe signs of lethality: Polyuria, severe dyspnea, severe tremors (muscle fasciculations), ataxia, seizures, severe lethargy (sluggishness, stupor, prostration).	Mainly septic shock

* The dose ranges used for both toxicants are below their respective LD_50_ for experimental purposes. DFP: Diisopropylfluorophosphate; LPS: Lipopolysaccharide; LD_50_: Median lethal dose; i.p.: Intraperitoneal injection; s.c.: Subcutaneous injection.

## Data Availability

The data presented in this study are available upon request from the corresponding author. However, they are not publicly available due to the Nova Southeastern University Dr. Kiran Patel College of Osteopathic Medicine’s privacy policy.
